# Complete Genome Sequence of *Corynebacterium kutscheri* DSM 20755, a Corynebacterial Type Strain with Remarkably Low G+C Content of Chromosomal DNA

**DOI:** 10.1128/genomeA.00571-15

**Published:** 2015-05-28

**Authors:** Christian Rückert, Andreas Albersmeier, Anika Winkler, Andreas Tauch

**Affiliations:** a Department of Biology, Massachusetts Institute of Technology (MIT), Cambridge, Massachusetts, USA; b Institut für Genomforschung und Systembiologie, Centrum für Biotechnologie (CeBiTec), Universität Bielefeld, Bielefeld, Germany

## Abstract

The complete genome sequence of the type strain *Corynebacterium kutscheri* DSM 20755 comprises 2,354,065 bp and 2,047 protein-coding genes. The mean G+C content of the chromosomal DNA is 46.46%, which is the lowest value detected so far in a member of the genus *Corynebacterium*.

## GENOME ANNOUNCEMENT

*Corynebacterium kutscheri* has been described as a commensal bacterium in mice and rats and has been detected frequently in the oral cavity of these rodents (1, 2), which may develop severe illness if they are immunocompromised or nutritionally deficient (3, 4). The only report of *C. kutscheri* causing an infection in humans is related to a 7-month-old girl with an infected right middle finger following a rat bite (5). Two previous reports of *C. kutscheri* infections in humans are doubtful, as the data did not provide a solid taxonomic identification of *C. kutscheri* in the clinical samples (6–8). Isolates of *C. kutscheri* from rodents are used in infection models of laboratory mice (9), whereas isolates from the marine environment and industrial sewerage have the potential to be applied in biodegradation of crude oil (10, 11) and in water pollution control by detoxifying heavy metal ions via biosorption (12). To establish the genetic background of this versatile corynebacterium, we sequenced the complete genome of the type strain *C. kutscheri* DSM 20755.

Genomic DNA of *C. kutscheri* DSM 20755 was obtained from the Leibniz Institute DSMZ. A shotgun DNA library was constructed by means of the Nextera DNA sample preparation kit (Illumina) and was sequenced in a × 250-nucleotide paired-end run using the MiSeq reagent kit v2 (500 cycles) and the MiSeq desktop sequencer (Illumina). Shotgun sequencing resulted in 2,817,451 paired reads and 457,240,592 detected bases. The Roche GS *de novo* Assembler software (release 2.8) was used to assemble the paired-read data, generating 16 scaffolds with 21 scaffolded contigs. Gaps in the genome sequence were bridged by adding 206,088 mate pair reads to the initial assembly. For this purpose, a 7-kb mate pair library was prepared with the Nextera mate pair sample preparation kit according to the gel-plus protocol and including a size selection of 500-bp inserts. The mate pair reads were obtained by DNA sequencing with the MiSeq reagent kit v3 (600 cycles). The gap closure step of this genome project was facilitated by Consed (version 26) (13). Gene recognition was performed with the Prodigal software (14) and the functional annotation of the detected protein-coding regions was supported by the IMG/ER pipeline (15).

The chromosome of *C. kutscheri* DSM 20755 has a size of 2,354,065 bp and comprises 2,047 protein-coding genes, 52 tRNA genes, and 4 rRNA operons. The mean G+C content of the chromosomal DNA is 46.46%, which is the lowest value detected in a corynebacterial type strain (16). This result is almost identical with the value (46.2%) described in 1983 by Pitcher, who used the thermal denaturation method to determine the DNA base composition of *C. kutscheri* (17). A mean G+C content below the defined range for *Corynebacterium* species (from 52 to 68 mol%) was found only in *Corynebacterium caspium* (49.7 mol%) and *Corynebacterium freiburgense* (49.8 mol%). The genome annotation indicated that the cell surface of *C. kutscheri* DSM 20755 possesses adhesive pili of the SpaGHI type, already detected by electron microscopy in 1976 (18).

### Nucleotide sequence accession number.

This genome project has been deposited in the GenBank database under the accession no. CP011312.
